# Increased AGE Cross-Linking Reduces the Mechanical Properties of Osteons

**DOI:** 10.1007/s11837-024-06716-x

**Published:** 2024-07-29

**Authors:** Ihsan S. Elnunu, Jessica N. Redmond, Yoshihiro Obata, William Woolley, David S. Kammer, Claire Acevedo

**Affiliations:** 1 Department of Mechanical Engineering, University of Utah, Salt Lake City, UT 84112 USA; 2 Institute for Building Materials, ETH Zurich, Laura-Hezner-Weg 7, 8093 Zurich, Switzerland; 3 Department of Biomedical Engineering, University of Utah, Salt Lake City, UT 84112 USA; 4 Department of Mechanical and Aerospace Engineering, University of California San Diego, Engineers Ln, San Diego, CA 92161 USA

## Abstract

The osteon is the primary structural component of bone, contributing significantly to its unique toughness and strength. Despite extensive research on osteonal structure, the properties of osteons have not been fully investigated, particularly within the context of bone fragility diseases like type 2 diabetes mellitus (T2DM). This study aims to isolate osteons from bovine bone, simulate the effects of increased advanced glycation end-products (AGEs) in T2DM through ribosylation, and evaluate the mechanical properties of isolated osteons. Osteons extracted from the posterior section of bovine femur mid-diaphysis were processed to achieve a sub-millimeter scale for microscale imaging. Subsequently, synchrotron radiation micro-computed tomography was employed to precisely localize and isolate the osteon internally. While comparable elastic properties were observed between control and ribosylated osteons, the presence of AGEs led to decreased strain to failure. Young’s modulus was quantified (9.9 ± 4.9 GPa and 8.7 ± 3 GPa, respectively), aligning closely with existing literature. This study presents a novel method for the extraction and isolation of osteons from bone and shows the detrimental effect of AGEs at the osteonal level.

**Supplementary Information:**

The online version contains supplementary material available at 10.1007/s11837-024-06716-x.

## Introduction

The increased fracture risk among adult patients with type 2 diabetes mellitus (T2DM) is a critical medical concern, evidenced by studies indicating a threefold increase in fracture risk in this population.^1–3^ In light of the escalating global diabetes epidemic, affecting one in ten adults worldwide, the need to unravel the intricate mechanisms contributing to diabetic bone fragility becomes increasingly urgent.^1^ Several other bone fragility diseases, including osteoporosis, are clinically characterized by loss of bone mass. In contrast to osteoporosis, T2DM is unique in that bone mass remains intact. Instead, it is affiliated with changes in bone quality.^4–8^ These alterations predominantly influence factors such as material properties and microstructural integrity.^4,7,9^ Bone derives its unique properties, strength, and fracture toughness (i.e., resistance to fracture) from its intricate hierarchical multiscale arrangement of mineralized collagen fibrils.^10,11^ These mineralized collagen fibrils assemble into lamellae, forming osteons characteristic of the cortical bone structure, with a central canal at their core. The hierarchical structure of bone allows it to repair, adapt, and withstand mechanical stresses, making it an exceptional biological material.^10–16^

At the microscale, osteons as the primary structural components in cortical bone, contribute to bone’s toughening mechanisms. Primary osteons are formed first in the periosteum region during bone growth. These immature osteons are later replaced by secondary osteons generated during internal bone remodeling. In this study, we will use the term "osteons" to specifically denote secondary osteons. Osteons are cylindrical features found in mature human and mammalian bone tissue, typically around 300 µm in diameter. They are formed of concentric lamellar layers wrapped around a central Haversian canal.^17–19^ At the interface with interstitial bone tissue, the secondary osteons are bordered with cement lines (e.g., highly mineralized boundaries) while primary osteons do not possess cement lines since they are not yet associated with bone remodeling. These osteon structures play a primary role in toughening bone by actively deflecting and twisting the crack path along the cement lines, dissipating energy and enhancing the bone’s resistance to failure.^20–23^ Given their important role in the microscale toughness of bone, it is essential to understand the function of osteons, particularly in the context of bone fracture-associated fragility disease.

To identify the determinants of differences in bone quality, it is necessary to consider all levels of the hierarchical structure of bone, including the collagen level. However, the hierarchical nature of deformation and toughening mechanisms is particularly challenging to investigate in bone. In addition to crack deflection at the microscale, toughness originates from the collagen’s capacity for deformation at the nanoscale.^24–29^ Within the context of T2DM, hyperglycemia, characterized by elevated blood glucose levels, leads to the formation of specific collagen cross-links known as advanced glycation end-products (AGEs).^30–32^ The accumulation of AGEs has been observed to stiffen the collagen by hindering collagen’s ability to deform, thereby altering whole bone’s mechanical behavior and making it more brittle.^5,6,27,33–40^ However, the independent influence of AGE cross-links on osteon’s toughness remains unclear.

Despite existing literature on osteonal mechanical behavior, research in this area remains limited both numerically and experimentally. The mechanical response of osteons under tension was examined in the 1960s to the 1980s, predominantly by a single research group.^41–43^ The numerical characterization of the mechanical behavior of osteons, including in tension, has been studied more recently.^44–48^ Therefore, the novelty of our work is crucial for bone research in terms of quantifying the mechanical properties of osteons experimentally. The previous work assumed the osteons grow perfectly straight and do not change in size along its length. Previous work also identified the osteons by looking at the top surface and then cutting and isolating the osteon in the middle of the sample without considering the possibility of missing the osteon. Our work ensures the osteon is present in the tested region by using a meticulous procedure of identification and isolation while also determining the impact of AGEs on these structures.

By investigating the intricate mechanisms of micro- and nanoscale in bone, this study aims to provide valuable insights into the links between AGE accumulation, bone microstructure, and bone quality. Here, we will test the hypothesis that, by isolating one single aspect of T2DM, AGE accumulation, the osteon’s post-yield mechanical behavior will be impaired. Understanding how collagen changes can impact the mechanical behavior of osteons is critical in determining the origins of fragility fractures in bone with AGE accumulation. To achieve this essential understanding, implementing a novel mechanical testing method on isolated osteons is a crucial step in understanding the effects of AGEs on the osteon’s capacity to deflect cracks and resist fracture, ultimately leading to either less tough or more fragile bone. Developing a novel mechanical test to evaluate isolated osteons is a crucial step to bridge this knowledge gap. This work will result in new data to identify the contribution of AGE content on osteon’s mechanical behavior and will shed light on the distinct pathophysiological mechanisms contributing to bone fragility in T2DM.

## Materials and Methods

### Study Design

The samples used in this study were extracted from the mid-diaphysis of bovine femurs. The grass-fed cow’s femur was cut and divided into four 40-mm-long sections. A slice from the cross-sectional surface was imaged to distinguish osteonal and plexiform bone sections. Examination under an optical microscope revealed that the majority of osteons were concentrated on the posterior side of the femur mid-diaphysis, consistent with prior research.^49–53^ Matchstick-like samples were cut and polished to dimensions of 1 mm $\times$ 1 mm $\times$ 20 mm under constant hydration, containing at least one osteon in their center. Following this isolation process, samples were imaged using synchrotron radiation micro-computed tomography (SRµCT) to obtain the osteon’s precise position and orientation. Using the 3D image, the samples were necked down to reveal only the osteons on a length of approximately 8 mm. To mimic a consequence of T2DM, samples were incubated in a ribose solution to increase the AGE content. Mechanical properties of the isolated osteon were measured using a tensile test.

### Osteon Identification, Extraction, and Polishing

Under an Olympus CX41 upright microscope equipped with a 14 MP microscope camera (AmScope MU1403), both surfaces of the cross sections were examined. Regions of interest were identified based on the presence of secondary osteons exhibiting hypermineralized osteonal boundaries (cement lines). Once identified, osteonal regions were marked on the cross-section and correlated with the original posterior bone section. This process facilitated the isolation of osteon-rich regions, which were retained for further processing. Both surfaces of these selected sections were then polished using an Ultra Tec polishing table. Subsequently, 2-mm-thick cross sections were cut from each polished side of the newly processed sections using the Allied Low-Speed Precision Saw, equipped with a diamond blade, under constant irrigation. The cut sides of these cross sections were then polished using the polishing table until a uniform thickness ($\approx$ 1.5 mm) was attained. Both faces of the cross sections were mirror polished and then once again taken to the microscope.

Both faces of the cross sections were examined to determine the angle at which osteons ran lengthwise within the sample. The angles of the osteons were measured about one of the long sides of the cross section using the camera’s digitally calibrated measurements. These angle measurements were then related to the regions of interest from which they were derived, providing a relative angle of osteon travel within the sample. The samples were secured to the Allied Low-Speed Precision Saw with the identified angle, utilizing a custom-made mounting fixture aligned with the predetermined angle. The grid of samples was subsequently moved to the Allied Manual Table Saw, where the cuts previously initiated were continued through the uncut bottom end, effectively separating each sample. After each separation, the samples were left in PBS to replicate physiological conditions. Subsequently, we polished each sample’s sides on the polishing table, ensuring that each side reached a uniform thickness, with a variation of no more than 0.05 mm. Two additional cross sections were prepared from each sample, with careful considerations made to maintain the orientation of the bone.

**Fig. 1:**
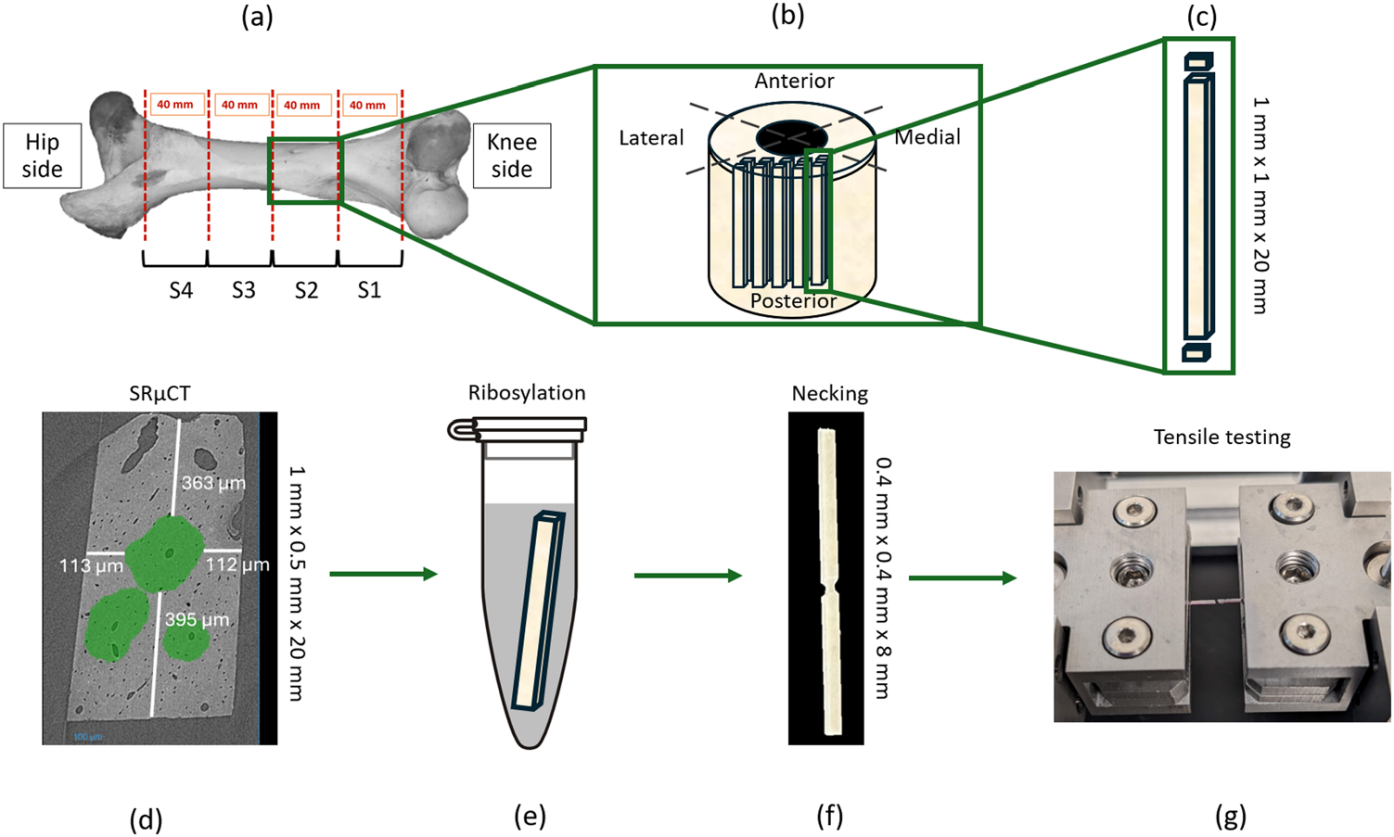
(a) A bovine femur was sectioned into four quadrants to localize regions with osteons. (b) From the posterior mid-diaphysis region, rich in secondary osteons, samples were cut for investigation because of high probability of osteon presence. (c) Cross sections of each sample were analyzed under a light microscope to evaluate the presence of osteons. (d) Samples most likely to have an intact osteon in their core were imaged using SRµCT to obtain precise position and orientation of the osteon. (e) After appropriate samples were selected, they were separated into control (*n* = 8) and ribosylated (*n* = 7) groups, where ribosylated samples were incubated in a ribose solution to increase the AGEs content. (f) Samples were necked down using a drill mill to isolate the osteon. (g) Samples were tested under a quasi-static tensile test to measure mechanical properties.

The upper and lower faces of the cross sections were examined for secondary osteons > 0.25 mm in diameter. The locations of these secondary osteons within each sample were recorded in relation to the sample’s overall structure. A minimum diameter of 0.25 mm was chosen for secondary osteons to ensure robust sample integrity as smaller samples were more susceptible to microcracks and fractures during the preparation phase. The sample preparation and osteon identification process can be seen in the schematic in Fig. 1.

### Microscale Imaging to Verify the Existence and Precise Location of the Osteon for Isolation

**Fig. 2:**
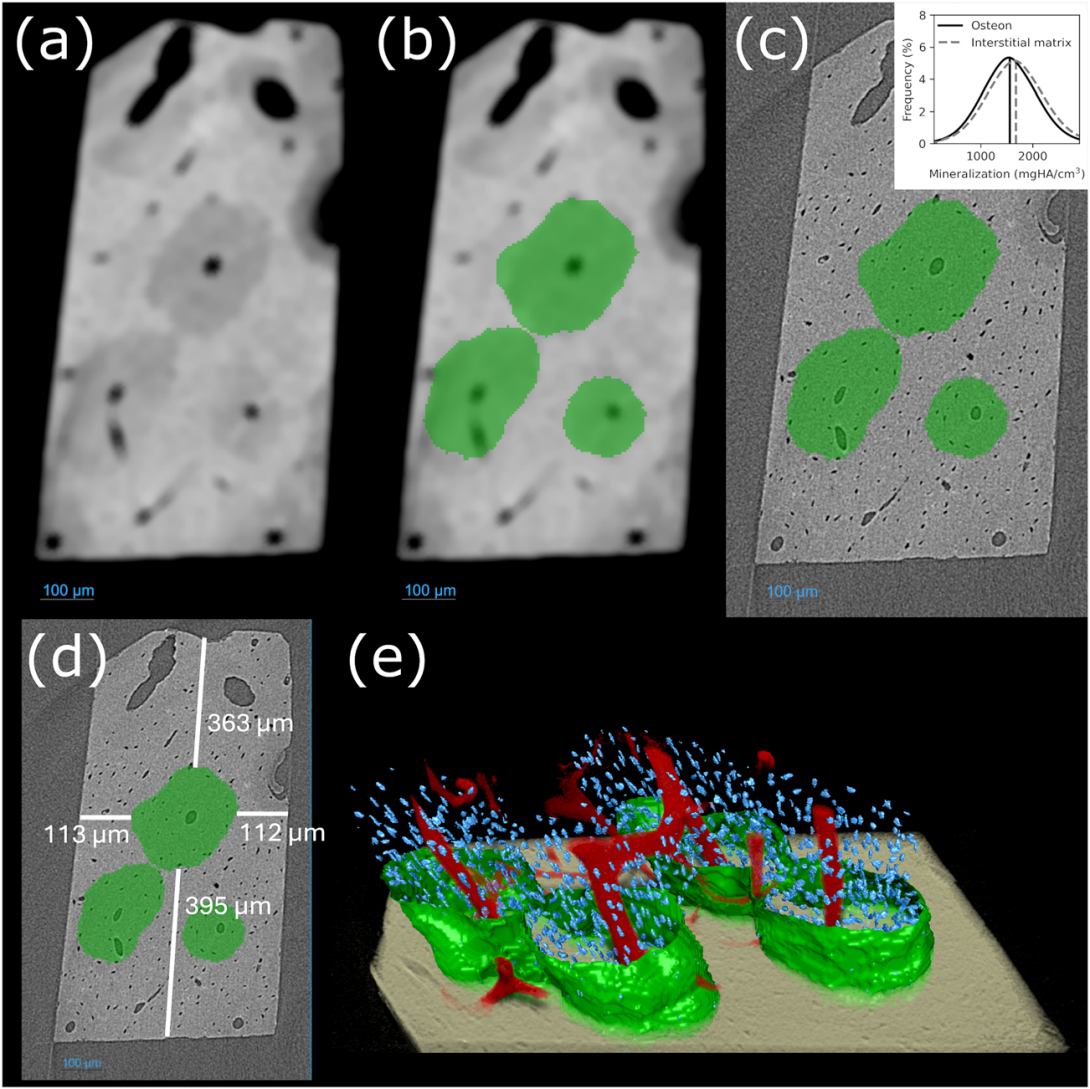
Synchrotron radiation micro-computed tomography (SRµCT) visualization of full osteons and aid in isolating them. (a) Osteons are first identified by image processing filters (Gaussian blur, median filtering, and brightness and contrast enhancement) to improve their contrast compared to the surrounding bone matrix. (b) These images are then used in combination with histogram equalization and Hessian operations to segment rough outlines of the osteons in bone. (c) The osteon region of interest is shown here overlayed on the original SRµCT image. (d) The exact location of the osteon is measured using the images and segmentation so this region of bone can be isolated for mechanical testing. (e) The osteon segmentation (green shell), osteocyte lacunae within the osteon region (blue), and the canals within the bone sample (red) are shown to illustrate the shape and microstructural contents of the osteon in three dimensions (Color figure online).

Samples of interest meeting the criteria for the presence of secondary osteons underwent microscale imaging to obtain precise internal measurements (*n* = 4). These samples were further polished to 1 mm $\times$ 0.5 mm before imaging. The microscale imaging was performed at the synchrotron microtomography beamline 8.3.2 at the Advanced Light Source in Berkeley, CA. Images were acquired with a beam energy of 24 keV with an exposure time of 50 ms and 985 projections to avoid deterioration of mechanical properties. Vertical stitching of three regions of interest of approximately 1.6 mm in height created a total region of interest of 4.8 mm from which to segment osteons. The pixel size of each image was 1.6 µm/pixel. To segment the osteons, images were downsampled by a factor of 3, and Gaussian and median filters were applied to the image to highlight the osteon features. Histogram equalization and 2D Hessian operations were then performed with manual region-of-interest tuning to obtain the final osteon segmentation. The SRµCT images were analyzed using Dragonfly (ORS) software to precisely locate the osteons and track their length as seen in Fig. 2. Once the orientation and location of an osteon had been assessed using the SRµCT images, the bone sample was necked down to dimensions of 0.4 mm × 0.4 mm × 8 mm using a Dremel rotary saw. The necked region was obtained by removing the mineral matrix from the four sides of the samples. This 0.16 mm$^2$ region was designated for testing and represented an isolated osteon within each sample (Fig. 1f).

### Ribosylate Samples to Mimic T2DM

Following sample preparation and extraction, the samples were separated into two groups: ribosylated (*n* = 7) and control (*n* = 8), mirroring one main effect of diabetes, i.e., increase in AGEs, and healthy bone conditions, respectively. Ribosylated samples underwent immersion in a 0.6M ribose solution, at 37°C, with regular exchanges of the ribosylation solution every 3 days. The ribosylation treatment lasted for 7 days, mirroring the pioneering work of Vashishth et al.^37^ Their research revealed AGE accumulation through in vitro ribosylation over the same duration, approximating AGE accumulation spanning 3 decades in individuals with T2DM.^39^

### Osteon Uniaxial Tensile Test

To evaluate the mechanical properties of the osteon, we conducted a tensile test on the osteons with a cross-sectional area of 0.16 mm$^2$ utilizing a Psylotech MicroTest System (Psylotech Incorporated, Evanston, IL, USA) until fracture. A 220-N load cell was used for testing with an 8-mm span between the grips. The uniaxial tensile tests were performed with a displacement speed of 833 nm/s. Due to the small size of the samples, and specifically the tested region, samples had to be carefully fixed in the clamps using grooved clamps and torqued at 0.8 Nm to avoid slippage and breakage. Samples were consistently hydrated by pipetting drops of PBS onto the tested region of all bone samples immediately prior to testing. Force displacement data were recorded during the tensile testing. Subsequently, we calculated the stress and strains. The strain to failure, $\varepsilon _{f}$, is defined as the maximum percent strain at failure. The work to fracture, WoF, is defined as the work per unit area to fracture the sample. All analysis was performed using Python.

### Fractography

The osteon fracture surface and the cross-section area and diameter were imaged and measured using a scanning electron microscope (SEM) (JEOL JSM-5910LV). The images of the crack path and the osteon’s fracture surface were captured with back-scatter electron mode at a voltage of 15–25 kV. The SEM examination allowed for a microstructural assessment of the transverse-fractured samples caused by the tensile testing failure. Afterward, the acquired SEM images were analyzed using ImageJ, an open-source image processing software.^54^ We used ImageJ to measure the area of the isolated osteon to accurately calculate the mechanical properties. To calculate the cross-sectional area, we used Gaussian and median filtering to enhance the contrast, manually selected the osteon and subtracted the background and canals from each cross-section, and finally outputted the area resulting from the isolated osteon selection using the Particle Analyzer plugin.

### Fluorimetric Assays to Quantify AGEs

After testing the samples, a fluorimetric assay was performed to assess AGE concentrations in both control and ribosylated specimens, as previously detailed.^55,56^ The section of the sample from the necked region to the clamp underwent a decalcification process, followed by hydrolysis. Decalcification involved immersing the samples in a 20% ethylenediaminetetraacetic acid (EDTA) solution, and hydrolysis was achieved through treatment with proteinase K (1 mg/ml for 3 h at 60°C). Fluorescence was directly read with an excitation wavelength of 370 nm and an emission wavelength of 440 nm, using a microplate reader (SpectraMax M2, Moleucular Device, USA). Fluorescence values were compared to a quinine sulfate standard and normalized to collagen content, calculated based on the wet weight. The fluorimetric assay was performed on a small segment from the tested necked region to consistently support and represent data from the same region used for mechanical properties.

### Statistical Analysis

After removing samples that slipped from the grips during mechanical testing, mechanical properties were calculated with eight control osteon samples and seven ribosylated osteon samples. Due to the unequal variances and sample sizes, a Welch’s t-test was performed with $\alpha$ = 0.05. Type II error was measured to assess the probability of having false-negative results.

## Results

### Ribosylated Osteons Have Significantly Increased AGEs Content

**Fig. 3:**
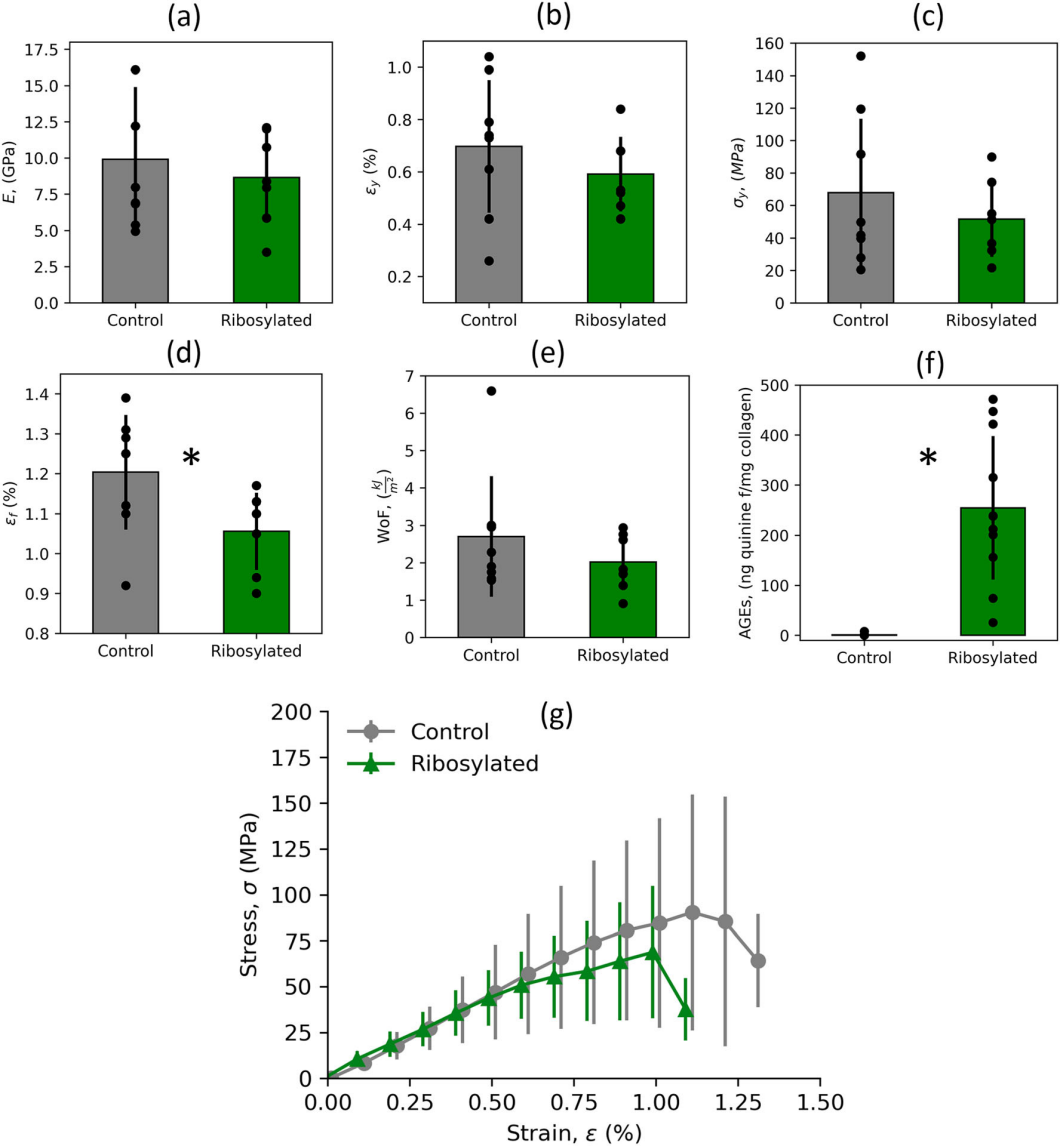
Mechanical properties in bovine bone’s osteons comparing control and ribosylated groups (*n* = 8 control, *n* = 7 ribosylated). Yield and pre-yield properties (a) Young’s modulus, (b) yield strain, and (c) yield stress were not statistically significantly altered by AGEs accumulation, whereas the post-yield property (d) strain to failure (*p* = 0.045 < 0.05) was statistically significantly lower for the ribosylated group. (e) work to fracture (Wof) (*p* = 0.34 > 0.05) was not significantly lower in the ribosylated group than in the control group. The WoF shows a trend of lower WoF in the ribosylated group. (f) AGE quantification for 7 days of ribosylation comparing control and ribosylated groups. The ribosylated group is statistically significantly more (*p* = 0.0001 < 0.01). Data are given as mean ± SD. (g) Tensile test stress-strain curve. The solid gray line indicates the mean stress-strain curve of the control group with the error bar denoting the standard deviation at interpolated values of 0.1 strain. Similarly, the mean of the ribosylated group is denoted by a solid green line with the error bar showing the standard deviation (Color figure online).

To evaluate collagen cross-linking and quantify AGE concentrations, a fluorimetric assay was implemented on both control and ribosylated specimens after mechanical testing. As expected, the 7-day ribosylation process significantly increases the concentration of AGEs in the ribosylated specimens compared to the control group (*p* = 0.0001 < 0.01), as illustrated in Fig. 3f. This marked 24-fold difference confirms the impact of ribosylation on AGE accumulation in the tested samples, providing valuable insights into the glycation-induced alterations in collagen cross-linking within the context of our study.

### SRµCT Imaging of Osteons Confirms that Interstitial Bone Tissue Is Stiffer Than Osteon Bone Tissue

As a critical component of osteon isolation, bone samples were imaged using SRµCT to confirm the existence and location of osteons running longitudinally through each bone as seen in Fig. 2. This segmentation for each scanned sample was used to isolate a single osteon for final mechanical testing. In addition to locating the exact positions of osteons, the segmentations were used to compare the mineral density of the osteonal tissue and the non-osteonal bone matrix. Osteonal tissue possessed a mean volumetric tissue mineral density (vTMD) of 1560 ± 520, with non-osteonal matrix having a mean vTMD of 1680 ± 530. Despite more noise present in these SRµCT images due to the scanning parameters accommodating low radiation dosage, lower vTMD is observed both visually through image processing and in calculated vTMD values (–7% decrease). Because vTMD values are correlated highly with Young’s modulus, this shows the change in material properties from osteonal tissue to the insterstial matrix that has been observed in the literature.^57^

### AGEs Impair Post-yield Properties While not Affecting Pre-yield Properties

Analysis of the tensile test results conducted on isolated bovine osteons revealed a distinct pattern in the response of pre- and post-yield properties to the accumulation of AGEs, as illustrated in Fig. 3. Examining the pre-yield properties, we observe that Young’s modulus, denoted as *E*, exhibited no statistically significant difference between the control and ribosylated groups. Similarly, at the yield point, both the yield strain, $\varepsilon _{y}$, and yield stress, $\sigma _{y}$, displayed no significant difference between the two groups. This behavior at and before the yield point suggests that the initial elastic behavior and the onset of plastic deformation in the osteons remained unaffected by the presence of AGEs. Interestingly, although not significant, there is a trend of decreases in elastic properties with ribosylation. While this could be due to variance in the samples tested, the consistent, small decrease in these properties suggests that ribosylation may have had a slight effect in decreasing even elastic properties in this study at the osteonal scale.

However, a distinctive shift in mechanical behavior emerged at the post-yield stage. The strain to failure, $\varepsilon _{f}$, demonstrated a significant reduction in the ribosylated group compared to the control ($-$12.3% decrease), indicating impaired ductility and structural integrity in the presence of AGE accumulation. While the WoF did not exhibit a statistically significant difference, a discernible trend was evident, indicating a lower WoF in the ribosylated samples (–25% decrease). This analysis highlights the relatively selective impact of AGEs on post-yield properties, signifying a vital measurement in understanding how glycation influences the mechanical behavior of osteons during fracture.

This decrease in energy dissipation is related to the decrease in ultimate tensile strength with ribosylation, as shown on the stress-strain curve in Fig. 3g. The ultimate strength in the control osteons surpassed that of the ribosylated osteons. This trend is visually evident after the end of the elastic region. However, it is noteworthy that, despite the observable trend, we found no statistical significance between the ultimate stress values of the control and ribosylated groups (*p* = 0.23 > 0.05).

**Fig. 4:**
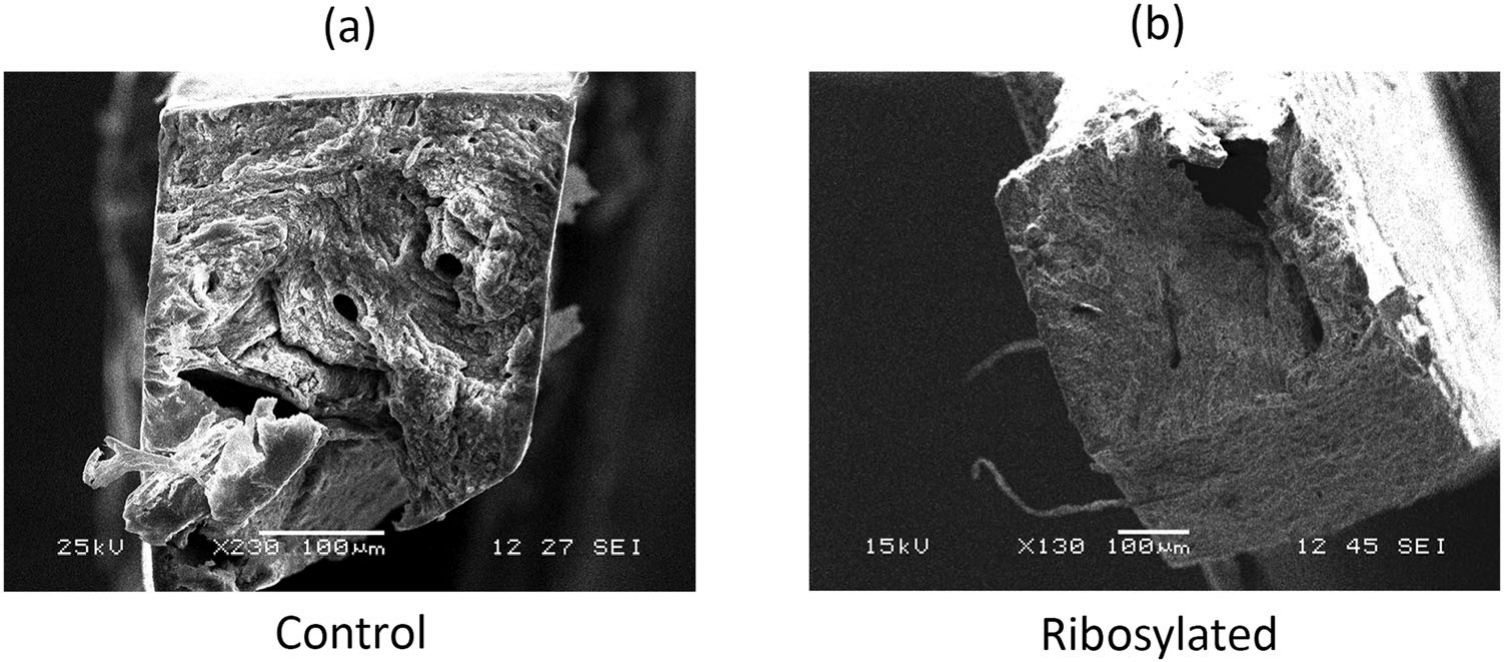
Scanning electron microscope (SEM) images of fracture surfaces of (a) control sample (b) ribosylated sample.

### AGEs Accumulation Leads to Low-Energy Fracture Behavior

Fracture surfaces obtained from the tensile test were observed using SEM (Fig. 4). These images confirm the change in toughness from mechanical results by visually showing a rougher surface (with loss of tortuosity and out-of-plane deflections) in the crack path of the control group fracture surface versus a much smoother fracture surface in the ribosylated group. This indicates that AGEs contribute to a low-energy and flatter crack path. This change in crack fracture surface further emphasizes the compromised toughness of the bone structure.

## Discussion

In this work, we conducted mechanical tests on isolated osteons, which are primary structural components found in both human and mammalian bone, and determined the influence of AGEs on their mechanical properties. The method of extracting the osteon using microscopy, honing in on the internal location using SRµCT, and isolating these samples into a testable, dog-bone-like sample has never been performed before to our knowledge. In the past, researchers have assumed that osteons traveled straight down after identifying them on a cross section.^41,58,59^ This assumption is flawed as shown by our investigation since the osteons have varying lengths and growth angles, which was observed in both optical microscopy and SRµCT as seen in Fig. 2e. Because much of the work detailing the testing of osteons was performed decades ago until just recently, this isolation method can be used and iterated on to improve the future mechanical testing of osteons. The unique osteon isolation method developed in this work is effective but involves the use of SRµCT for osteon location in 3D, which is not readily available to all. Augmenting this method with a more readily available micro-CT machine that can still image osteons discernibly from surrounding tissue could improve the accessibility of this method.

Our hypothesis that AGE accumulation impairs the osteonal post-yield properties is validated based on our results in Fig. 3. A notable shift in mechanical behavior became apparent during the post-yield stage, with the ribosylated group exhibiting a significant reduction of 12.3% in the strain to failure, $\varepsilon _{f}$, compared to the control. This reduction signifies impaired ductility and compromised structural integrity in the presence of AGEs accumulation. Despite the lack of statistical significance in the work to fracture, the discernible trend of 25.2% reduction might suggest that a lower work to fracture in the ribosylated samples may become evident with a larger sample size. Indeed, the type II error is relatively high (0.87), indicating a high probability of failing to detect a difference when one actually exists. Increasing the sample size and reducing the variability by controlling additional factors beyond just the type of osteons would help decrease the probability type II errors. These findings are substantiated by Fig. 4, where the control group’s fracture cross section exhibits a rougher surface, while the ribosylated group displays a much smoother fracture cross section—providing visual confirmation that AGEs contribute to bone fragility by inducing a brittle fracture mode. Our findings are consistent with prior research.^42,48^ Control samples exhibit higher strain to failure and work to fracture of 1.2 ± 0.14 and 2.70 ± 1.57 $kJ/m^2$, respectively, compared to ribosylated strain to failure and work to fracture of 1.06 ± 0.093 and 2.019 ± 0.71, respectively. These results correspond to reduced trends observed in previous studies by Ascenzi et al.^42^ and Marty et al.^48^ The reducing trend of strain to failure, $\varepsilon _{f}$, with aging in osteons is observed where Ascenzi et al.^42^ reported percent elongation at the breaking point (i.e., $\varepsilon _{f}$) ranging from 6.84$-$20.62% in young adult humans to 7.68$-$14.28% in old adult humans. In the theoretical model by Marty et al.,^48^ osteons break at 1.18% strain in young adults compared to 0.95% strain in old adults. Furthermore, the theoretical model results closely match our control results, differing by only 1.7%. Similarly, the results of theoretical model closely align with our ribosylated results, showing an 11% difference. Regarding work to fracture, our findings reflect the trends observed in the literature for cortical bone. This includes studies conducted in a T2DM rat model, where a statistically significant difference between T2DM and control samples was observed.^56^

Investigating the stress-strain curves of isolated bovine osteons provided insights into the mechanical response with increased AGEs concentrations. The mean curves shown in Fig. 3g, accompanied by the standard deviation, revealed a noticeable trend. Specifically, the ultimate stress in the control group surpassed that of the ribosylated group, suggesting a decrease in the osteon’s mechanical behavior and ultimate stress due to AGE accumulation. This visual distinction in the stress-strain profiles raises questions about the potential implications for bone strength and fracture resistance. However, it is crucial to interpret these findings with caution, as the observed trend did not reach statistical significance (*p* = 0.23). This lack of statistical significance encourages additional exploration and consideration of other factors that might contribute to the observed differences in ultimate stress between the control and ribosylated groups, improving our understanding of the complex relationship between AGE accumulation and osteon mechanical properties. Our ultimate stress results for the control group are consistent in trends and results with previous work testing osteons in tension for ox and humans.^41–43^ Our findings indicate a control ultimate tensile strength of 93.75 ± 50.30 MPa, falling within the range of 85–120 MPa reported by Ascenzi et al.^42^ for young human osteons. The similarity between the ultimate tensile strength of bovine bones used in our study and the young human bones investigated by Ascenzi reaffirms that bovine specimens are appropriate for osteonal mechanical assessments. However, our ribosylated results, 64.58 ± 28.73 MPa, differ in the ultimate tensile strength for old humans, 85–110 MPa. This change is possibly attributed to our method of isolation being more accurate, which showed that AGEs weakened the osteonal post-yield properties in addition to us testing bovine osteons while they tested human osteons. Bovine bone has been shown to be an appropriate model for measuring biomechanical properties of human bone.^60^ Using bovine bone offers the advantage of minimizing confounding factors that could potentially result in the differences observed in the ultimate tensile strength between human osteons and bovine bone. Comparing our ultimate strength results to the multiscale model by Marty et al.,^48^ we find consistent results where our control data lie in their normal distribution that is centered at 113 MPa for young adults; however, our ribosylated results are lower than their mature adult results of 106 MPa, which is possibly because of biological variance and sample size. Looking at rib cortical bone results, we can see that our results lie in their range of 36.2–134 MPa for ages 60–99.

The investigation of pre-yield properties, including Young’s modulus (*E*), yield strain ($\varepsilon _{y}$), and yield stress ($\sigma _{y}$), revealed no statistically significant differences between the control and ribosylated groups. Our elastic modulus results, *E*, 9.90 ± 4.89 GPa and 8.65 ± 2.99 GPa for control and ribosylated groups respectively, match results reported by Ascenzi et al.^42^ Their results report the young human osteonal elastic modulus in the range of 4–15 GPa, while the old human osteonal modulus showed a slight decrease to a range of 4–11 GPa. While AGEs are not typically thought to affect the elastic properties of bone, this drop in the elastic modulus in their elderly human osteons may be due to increased AGEs concentrations in older humans, as we also observed a slight drop in elastic modulus with ribosylation. While this slight change may be observed, the lack of statistical significance and high variance in this parameter indicate that it is more likely that AGEs have very little effect on the elastic properties of the osteon. Multiscale theoretical model results of osteons by Marty et al.^48^ for mature and elderly humans showed no change in their elastic modulus, which also aligns with our experimental results, indicating no statistical difference. On the larger scale of cortical bone, the Young modulus was found to be higher in both middle-aged and mature humans compared to control and ribosylated results. Specifically, cortical bone showed a 52.5% higher modulus than our ribosylated osteons.^14^ Similarly, middle-aged human cortical bone indicated a 46.5% higher modulus compared to our control samples.^14^

In addition, looking at our results at the yield points, $\varepsilon _{y}$, and $\sigma _{y}$ in Fig. 3b, c, respectively (Tables S1–S2) (see online supplemental material), we have similar results where no statistical significance is reported in the literature between control and ribosylated samples as shown by work done by Willett et al.^61^ and in the theoretical model.^48^ The decrease in the osteonal properties compared to cortical bone properties could be attributed to isolating the osteon when testing. This is likely due to cortical bone’s mechanical properties as a whole being the product of multiple osteons, more mineralization, and interstitial bone; the interactions of all these factors contribute to cortical bone’s properties. The results from this study for *E*, $\varepsilon _{y}$, and $\sigma _{y}$ align with our established understanding that AGEs do not impact these pre-yield properties.^37,39,62,63^ The lack of impact on the pre-yield properties is explained by cross-linking, which plays a role in the plastic region through a collagen sliding and breaking mechanism, a fundamental intrinsic toughening mechanism.^24,35,64^ Interestingly, although there was no statistical significance, our results showed lower elastic properties with ribosylation, as seen in Fig. 3a, b and c and Tables S1–S2, indicating that if there is a small, not significant effect on elastic properties, it could be only on the smaller scale of the osteon, not the whole cortical bone.

Utilizing a fluorimetric assay on both control and ribosylated specimens allowed for the comprehensive assessment of collagen cross-linking and the quantification of AGE concentrations. The analysis exposed a significant elevation in AGE levels within the ribosylated specimens compared to the control group (*p* = 0.0001), as represented in Fig. 3f. Despite important variability, our results are consistent with others in literature^55,65,66^ where the number of AGEs is higher in ribosylated than in control bone. The observed differences in the mechanical properties contribute to understanding how one effect of T2DM, namely the accumulation of AGEs, affects the mechanical resistance of single osteons. Previous studies showed that AGE accumulation can lead to decreased whole bone mechanical properties^37,64,67,68^ without differentiating at which length scale this change was. Moreover, our study exclusively analyzes the impact of AGE accumulation, whereas T2DM affects not only collagen but also microstructural parameters such as canals and osteocyte lacunae.^56^

While our study provides crucial insights into the influence of AGE accumulation on the mechanical properties of isolated osteons, several limitations merit consideration. The challenge of isolating samples for testing arises from the inherent branching pattern of osteons, which do not uniformly follow identical angles and exhibit variations in diameter along their length. Additionally, limitations are associated with the selection of osteons for testing, as the brittleness of bone at smaller scales constrained us to test only larger osteons. This constraint was necessitated by the challenge of securely fixing samples into the micro-tester’s clamps without the risk of breakage, resulting in a range of tested osteon diameters from 250 µm to 300 µm. The secondary osteons in bovine femurs have a range of 52–297 µm.^69,70^ Furthermore, as seen in Fig. 1d, the osteons are consistent with the range reported in the literature.

Another common limitation in bone mechanics is the high standard variation, evident in our results as shown in Fig. 3 and in the supplemental material for the individual samples’ mechanical properties data points. The biological variation and the low sample size resulted in a high risk of having false-negative results (type II error). Osteons work together to make up the properties of the bone; therefore, testing one isolated osteon may not reflect all the osteons in that region. Lastly, testing the osteon in the transverse direction is extremely important for fully characterizing the osteon. Because the current work focused greatly on refining the technique of osteon extraction, the transverse direction was not tested for material properties; however, testing this orientation of osteons in the context of ribosylation would assist in developing a complete understanding of the effects of T2DM on the osteonal unit in bone in the future.

## Conclusion

In conclusion, our innovative approach of subjecting isolated osteons to mechanical tensile testing has shown the impact of AGEs on osteons in bone. The investigation confirmed that AGE accumulation predominantly impairs post-yield properties in osteons, as observed in the significant reduction of strain to failure ($\varepsilon _{f}$) in the ribosylated group compared to the control. This compromised ductility in the presence of AGEs highlights the intricate interplay between glycation and the mechanical behavior of osteons during fracture events. Examining the stress-strain curves revealed a discernible trend, with the control group visually exhibiting higher ultimate stress and lower work to fracture than the ribosylated group. Although these trends did not reach statistical significance, they prompt further exploration into potential contributing factors. The distinct fracture patterns observed using SEM imaging provide a visual confirmation that AGEs induce a brittle fracture mode, emphasizing their role in bone fragility. These findings enhance our understanding of glycation-induced alterations in collagen cross-linking, providing valuable insights into the complex relationship between AGE accumulation and osteon mechanical properties.

## Supplementary Information

Below is the link to the electronic supplementary material.

Supplementary file 1 (pdf 0 KB)
