# Supplementary material for: Increased AGE Cross-Linking Reduces the Mechanical Properties of Osteons
Source: JOM (1989). 2024 Jul 29;76(10):5692–702. doi: 10.1007/s11837-024-06716-x (PMC11417058; doi:10.1007/s11837-024-06716-x)
Supplement: Supplementary file 1 — (pdf 0 KB) [file 11837_2024_6716_MOESM1_ESM.pdf]

Increased AGE cross-linking reduces the mechanical properties of osteons

Ihsan S. Elnunu[1], Jessica N. Redmond[1], Yoshihiro Obata[1,4], William Woolley[1,4], David Kammer[2], and Claire Acevedo*[1,3,4]

[1]*Department of Mechanical Engineering, University of Utah, Salt Lake City, 84112, UT, USA*
[2]*Institute for Building Materials, ETH Zurich, Laura-Hezner-Weg 7, 8093 Zürich, Switzerland*
[3]*Department of Biomedical Engineering, University of Utah, Salt Lake City, 84112, UT, USA*
[4]*Department of Mechanical and Aerospace Engineering, University of California San Diego, Engineers Ln, San Diego, 92161, CA, USA*

Overview

This document contains the details and results related to:

- Mechanical properties per sample
- AGEs per sample

*Corresponding author. Email: claire.acevedo@gmail.com

Mechanical properties for each sample

Sample	Group	Yield Stress (MPa)	Yield Strain (%)	Young Modulus (GPa)	Ultimate Stress (MPa)	WoF (kJ/m^2)	Strain to Failure (%)
S2	Control	41.88	0.99	4.94	46.96	1.75	1.39
S16	Control	27.94	0.42	6.90	49.46	1.58	1.25
S17	Control	119.47	1.04	12.21	127.08	3.00	1.12
S19	Control	49.82	0.73	6.82	82.17	2.28	1.31
N12	Control	152.00	0.74	18.90	207.00	6.60	1.29
N13	Control	91.60	0.61	16.09	102.00	2.96	0.92
N8	Control	20.52	0.26	7.97	84.80	1.90	1.10
R14	Control	39.80	0.79	5.39	50.50	1.53	1.25
L11	Ribose	21.70	0.52	3.50	27.80	0.91	1.13
L31	Ribose	54.90	0.42	12.11	63.40	1.82	0.94
L41	Ribose	89.90	0.68	12.01	118.00	2.94	1.10
N4	Ribose	32.40	0.53	5.85	41.90	1.39	0.90
N7	Ribose	36.80	0.47	7.94	52.80	2.61	1.17
R13	Ribose	74.30	0.68	10.74	93.30	2.76	1.10
R41	Ribose	51.50	0.84	8.37	54.90	1.70	1.05

Table S1: Mechanical properties in each samples of bovine bone's osteons comparing control and ribosylated groups

AGEs data per sample

Sample	Group	AGEs (ng quinine/mg collagen)
R14	Control	8.09
L73	Control	1.62
N11	Control	0.00
R24	Control	0.00
L83	Control	0.43
L62	Control	1.21
L63	Control	0.00
N13	Control	0.00
N6	Control	0.00
N8	Control	0.00
N12	Control	0.00
L12	Ribose	212.64
R13	Ribose	156.17
N7	Ribose	239.31
L11	Ribose	74.27
R04	Ribose	238.29
N5	Ribose	200.94
L31	Ribose	315.12
R03	Ribose	471.60
N4	Ribose	422.05
L41	Ribose	447.17
R41	Ribose	25.48

Table S2: AGEs Quantification per sample for seven days of ribosylation comparing control and ribosylated groups
